# Case report: an unusual long-term evolution of a diffuse midline glioma, H3K27 altered

**DOI:** 10.3389/fonc.2025.1480247

**Published:** 2025-02-10

**Authors:** Michael Griessmair, Claire Delbridge, Claus Zimmer, Eva Mayr, Arthur Wagner, Julian Canisius, Marie-Christin Metz, Benedikt Wiestler

**Affiliations:** ^1^ Dept. of Neuroradiology, Klinikum rechts der Isar, Munich, Germany; ^2^ Department of Diagnostic, Interventional, and Pediatric Radiology, Inselspital Bern, University of Bern, Bern, Switzerland; ^3^ Department of Pathology, Technical University Munich, Munich, Germany; ^4^ Dept. of Neurosurgery, Klinikum Rechts der Isar, Munich, Germany; ^5^ TranslaTUM, Technical University of Munich, Munich, Germany

**Keywords:** H3K27M, tumorigenesis, advanced imaging, 850K methylation, WHO CNS classification 2021

## Abstract

**Background:**

As diffuse midline glioma, H3K27 altered, is a rare tumor entity with poor prognosis and few therapeutic options, only little is known so far about the genetic factors that influence tumorigenesis and the course of tumor development.

**Presentation:**

We present the case of a 38-year-old female patient who suffered from nausea, fatigue, and intermittent walking impairment, which developed over the course of four weeks. Initial MRI showed an irregularly shaped, contrast-enhancing tumor around the third ventricle with central necrosis, most likely originating from the right thalamus. The patient underwent biopsy, followed by microsurgical resection with molecular analysis. Molecular neuropathology revealed the diagnosis of diffuse midline glioma with a H3K27M mutation WHO (World Health Organization) CNS (central nervous system) grade 4. Interestingly, MR imaging conducted for migraine diagnosis 6 years ago in retrospect already showed a small, nodular T2w hyperintense lesion in the right thalamus.

**Conclusion:**

Despite a more precise, molecularly driven classification of pediatric HGG (high-grade glioma) in the 5th edition of the WHO classification of CNS tumors, many genetic factors influencing the biological tumor development as well as the precise molecular evolution of tumors remain unclear. Given the highly aggressive clinical course of these tumors, with a median overall survival around 16 to 18 months, our report of a (presumable) precursor lesion years before clinical manifestation point towards a complex, multi-stage evolution of this tumor entity. Better understanding this molecular cascade might help to identify novel targets for individualized therapies.

## Background

With the release of the fourth WHO (World Health Organization) classification for CNS (central nervous system) tumors in 2016, molecular markers were integrated into the diagnosis of brain tumors for the first time. For example, determining the diagnosis of an oligodendroglioma was made possible based on the presence of 1p/19q codeletion irrespective of a potential astrocytic histomorphology (1). Due to the increasing knowledge about the influence of molecular aberrations on tumor biology, some tumors were further specified, and new entities were created in the 5th edition of the WHO classification for CNS tumors 2021 (2). In this way, the differentiation of higher-grade gliomas into pediatric and adult types is also possible for the first time due to increasing understanding about their biological and genetic differences (3, 4). The higher-grade gliomas of pediatric type can be further divided into four subtypes: diffuse midline glioma, H3 K27 altered; diffuse hemispheric glioma, H3 G34 mutant; diffuse pediatric-type high-grade glioma, H3 wildtype; and IDH wildtype; infant-type hemispheric glioma (2).

Multiple sequencing analyses revealed an accumulation of two somatic point mutations in histone variants H3.3 and H3.1, resulting in an exchange of lysine to methionine at position 27 (5, 6). In healthy cells, H3K27-specific histone methyltransferase EZH2 (enhancer of zeste 2 polycomb repressive complex 2 subunit), part of the PRC2 (Polycomb Repressive Complex 2) catalyzes trimethylation of H3K27, which leads to gene repression via epigenetic regulation. This trimethylation is no longer possible due to the exchange of lysine to methionine, which leads to hypomethylation of the histone and thus to epigenetic dysregulation of oncogenes and tumor suppressor genes, and subsequently oncogenesis (7–9). It is well known that these gliomas are located in midline structures (thalamus, pons mesencephalon, cerebellum and spinal cord) and are associated with poor overall survival, because of limited surgical and medical therapies and the high biological aggressiveness of these tumors (10). Interestingly, individual cases of benign pilocytic astrocytomas (WHO CNS grade 1) with an H3K27 mutation have also been described in the literature (11). Hochart et al. showed a case of a spinal pilocytic astrocytoma, which showed secondary malignant progression to a glioblastoma, defined by old WHO tumor classification, after 10 years of stable course. Remarkably, an H3K27 mutation was detected in both the initial and secondary resection of this tumor. However, an additional TP53 mutation was detected in the second resection. Thus, it was postulated that the H3K27 mutation was the first step and TP53 the second step to malignancy (12).

Overall, H3K27-mutant diffuse midline gliomas are a relatively new entity, with their etiology and tumor biology poorly understood. Reporting this case is critical because it offers insights into possible molecular mechanisms that may drive the transformation from a low-grade to a high-grade glioma. This paper contributes to the growing body of evidence that specific molecular alterations, like the H3K27 mutation, play a pivotal role in tumor biology and progression. It underscores the importance of long-term monitoring and molecular analysis in patients with pilocytic astrocytomas, potentially influencing future diagnostic and therapeutic strategies.

## Case presentation

### Diagnostic

#### Anamnesis

A 38-year-old female patient presented with increasing nausea with vomiting, fatigue, and intermittent walking disorder over the last four weeks. Two weeks prior, the patient received gallbladder surgery. The patient herself had attributed her symptoms to this prior intervention. As a pre-existing condition, the patient reported suffering from migraine for several years. A long-term medication was denied. The family history was negative for cancer.

#### Physical examination

The examination did not reveal any neurological deficit. The patient appeared awake and responsive. There were no signs of anisocoria, urinary incontinence, or spastic movement disorders.

#### Imaging

**Figure 1:**
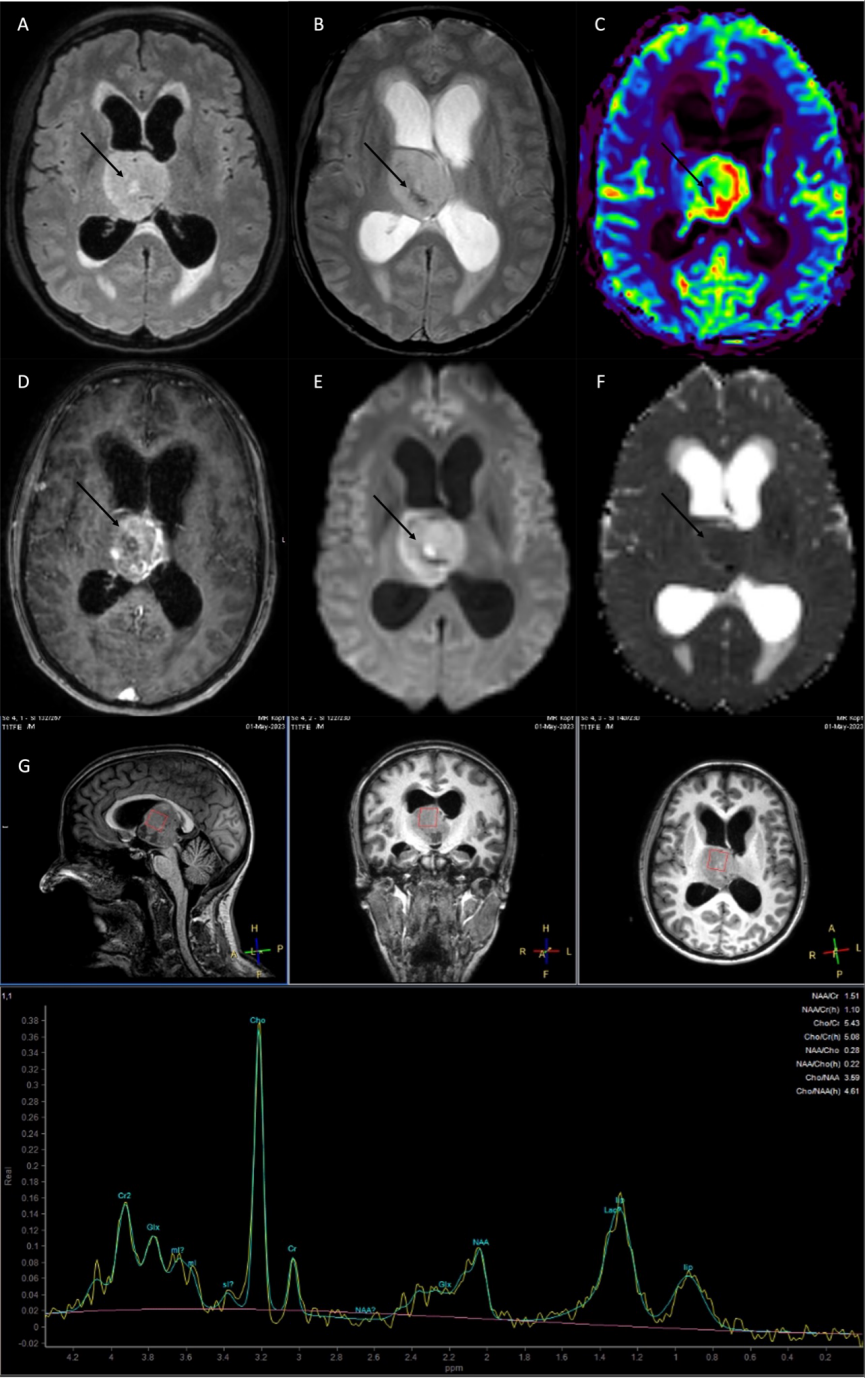
MRI at current presentation, showed a diffuse midline glioma H3K27 altered, most likely originating from the right thalamus with obstruction of the 3rd ventricle and consecutive CSF flow impairment. **(A)** FLAIR (fluid-attenuated inversion recovery), **(B)** T2*w, C: rCBV (relative cerebral blood volume), **(D)** T1w with contrast agent, **(E)** DWI (diffusion-weighted imaging), **(F)** ADC (apparent diffusion coefficient). **(G)** MR spectroscopy high levels of Cho (choline), lip (lipids), lac (lactate), and a decrease in NAA (N-acetylaspartate) and Cr (creatine). The tumor showed an irregular contrast-enhancement, with areas of intratumoral susceptibility [arrow in **(B)**], hyperperfusion [arrow in **(C)**], and low ADC values indicating high cellularity [arrow in **(F)**].

3T MR imaging (Philips Achieva) revealed a well-circumscribed, solid T2w-/FLAIR-hyperintense tumor with irregular contrast-enhancement and central necrosis around the third ventricle, presumably originating from the right thalamus. Given the tumor’s location and mass effect, consecutive CSF circulation impairment with dilated lateral ventricles and transependymal edema at the posterior and anterior lateral ventricles was seen. Central signal extinctions in the T2*w susceptibility sequence indicated the presence of single intra-tumoral calcifications or small hemorrhages, which could reflect the tumor biology (13). Advanced imaging showed tumor areas with high perfusion (CBV, cerebral blood volume) and low ADC (apparent diffusion coefficient) values, indicating the presence of angiogenesis and areas of high cellularity, two findings that are highly suggestive of a biologically aggressive, malignant tumor (14) (**Figure 1**). MR-spectroscopy showed a typical spectrum for high-grade glioma, with an increase in choline, lipids, and lactate and a decrease in creatine and NAA (N-acetylasparate) (**Figure 1**). There were no other pathological findings distant from the main finding.

### Histopathological and molecular analysis

**Figure 2:**
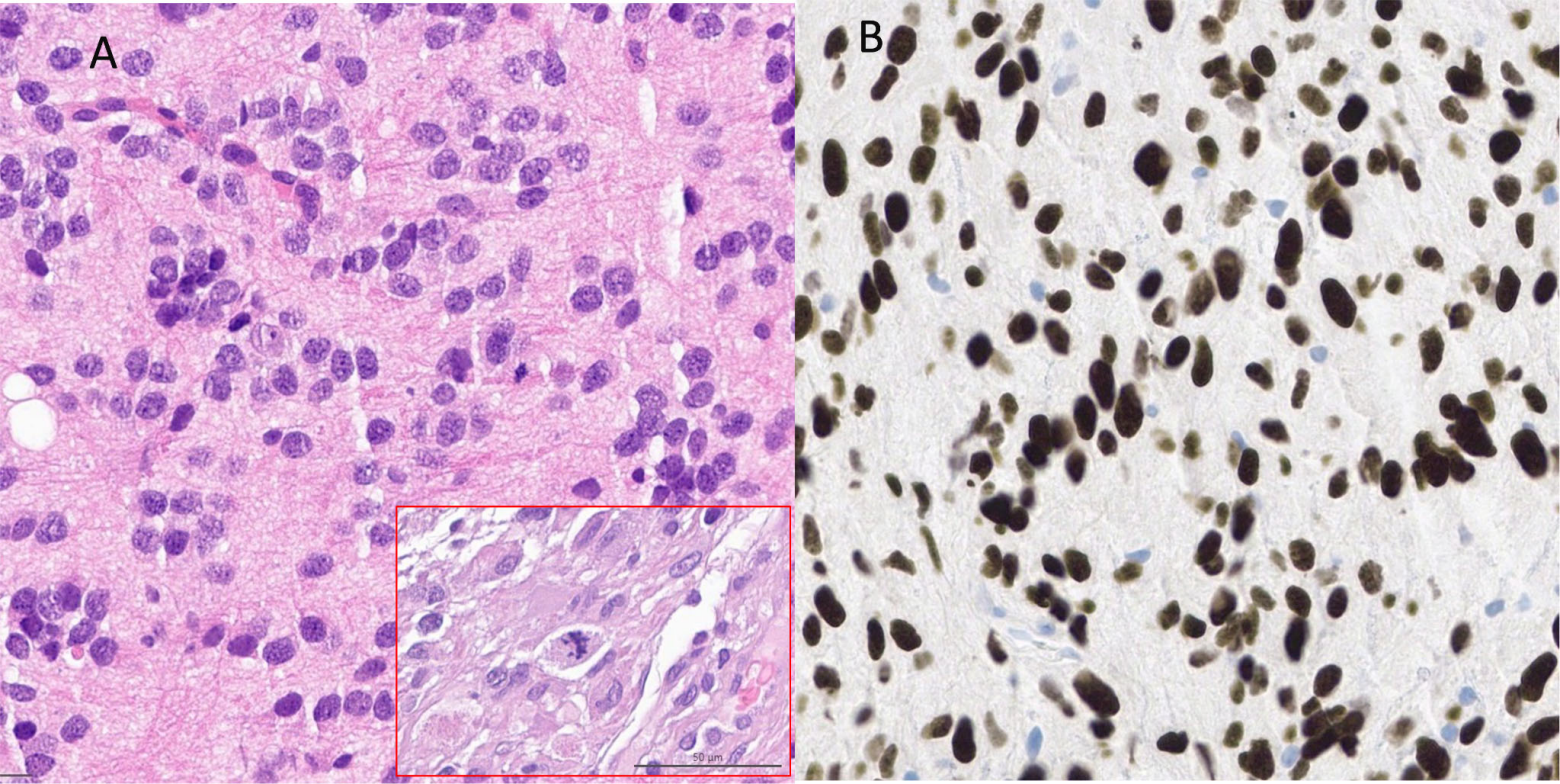
**(A)** HE staining showed diffuse infiltrating tumor with small monomorphic cells with predominantly roundish, partly oval, rather hyperchromatized, slightly enlarged nuclei with somewhat coarsened chromatin. Mitoses were clearly visible [shown in the red frame in **(A)**] **(B)** Immunohistochemistry reveals strong nuclear positivity for H3K27M.

**Figure 3:**
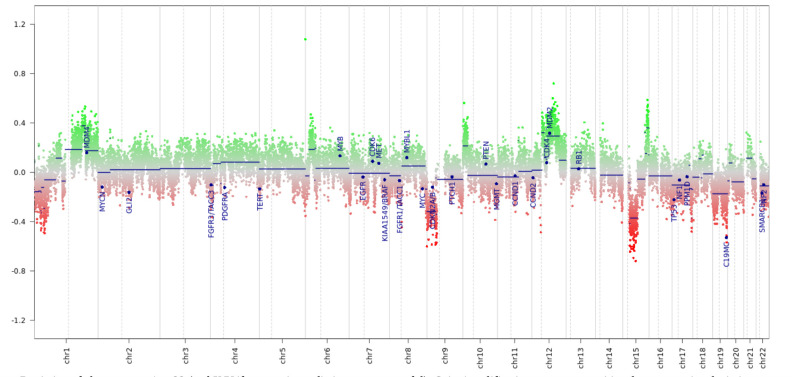
Copy number variation profile from the epigenome-wide methylation data. The tumor showed a CNV profile typical for biologically malignant gliomas, including losses on chr8 and gains on chr1, suggestive of chromosomal instabilities.

Initially, a stereotactic biopsy (Varioguide) was performed. Microscopy showed brain tissue with a diffusely infiltrating, very cell-rich glial tumor. The tumor cells were relatively small and monomorphic with predominantly oval, rather hyperchromatic, slightly enlarged nuclei, with somewhat coarsened chromatin. The cellular borders were blurred. Mitoses were readily visible (**Figure 2A**). The immune profile of the tumor cells showed the following results: H3K27K (nuclear strong positive) (**Figure 2B**), GFAP (positive), MAP2 (positive), IDH1 (no mutation), Ki-67 (focal up to 10%), ATRX (nuclear expression obtained), p53 (strong positive, no continuous accumulation), Olig2 (strong positive), ALDH (negative, surrounding glia positive), EGFR (negative). In the second step MRI-navigated microsurgical tumor resection was performed, and histopathological and molecular analysis with 850K methylation was done. Exact CNV (copy number variation) profile is shown below (**Figure 3**). Histological and immune profile revealed the same results as described above. The methylation analysis confirmed the diagnosis of a diffuse midline glioma H3K27M (Brain tumor classifier DKFZ, v11b4, Calibrated score: 0.92), nuclear expression of ATRX, no deletion in CDKN2A/B and non-methylated MGMT promoter.

The molecular profile strongly supported the diagnosis of H3K27M-mutant diffuse midline glioma, highlighted by strong H3K27K positivity confirming the mutation, a diffuse and highly cellular infiltrative pattern, and positivity for Olig2, GFAP, and MAP2 indicating glial differentiation. The high Ki-67 index of up to 10% suggested significant proliferation, and the methylation profile confirms the diagnosis with a calibrated score of 0.92. Notable deviations include retained ATRX expression, which is atypical for H3K27M gliomas, strong p53 positivity without continuous accumulation, and the absence of CDKN2A/B deletion, which is uncommon in this tumor type.

#### Differential diagnosis

Considering the signal behavior and the assumption that the tumor originated from an intraventricular site, a central neurocytoma could be considered as a differential diagnosis. The patient’s age and clinical symptoms were consistent with this entity; however, the multicystic component typically associated with neurocytomas is absent. Another differential diagnosis was an oligodendroglioma, which would align with the irregular barrier disruptions, central necrosis, and calcifications observed. A pilocytic astrocytoma was also a possibility; however, the absence of typical cystic areas with partially contrast-enhancing cyst walls makes this diagnosis less likely. Additionally, since Diffusion Weighted Imaging (DWI) demonstrated high intensity, an embryonal tumor should also be considered as a potential differential diagnosis.

#### Imaging 6 years ago

Interestingly, the same patient had already undergone MRI imaging 6 years ago as part of a migraine assessment. Retrospectively, a small FLAIR-hyperintense/T2w homogenous (~ cortical T2w signal intensity) lesion (maximal coronal diameter 8x4mm, axial 10x4.5mm) on the right thalamic side in the medial prefrontal thalamus adjacent to the 3rd ventricle with perhaps a slightly space-occupying component with mild displacing effect against the adjacent right internal cerebral vein could be delineated and without clearly defined boundaries, which could indicate early glioma growth.

**Figure 4:**
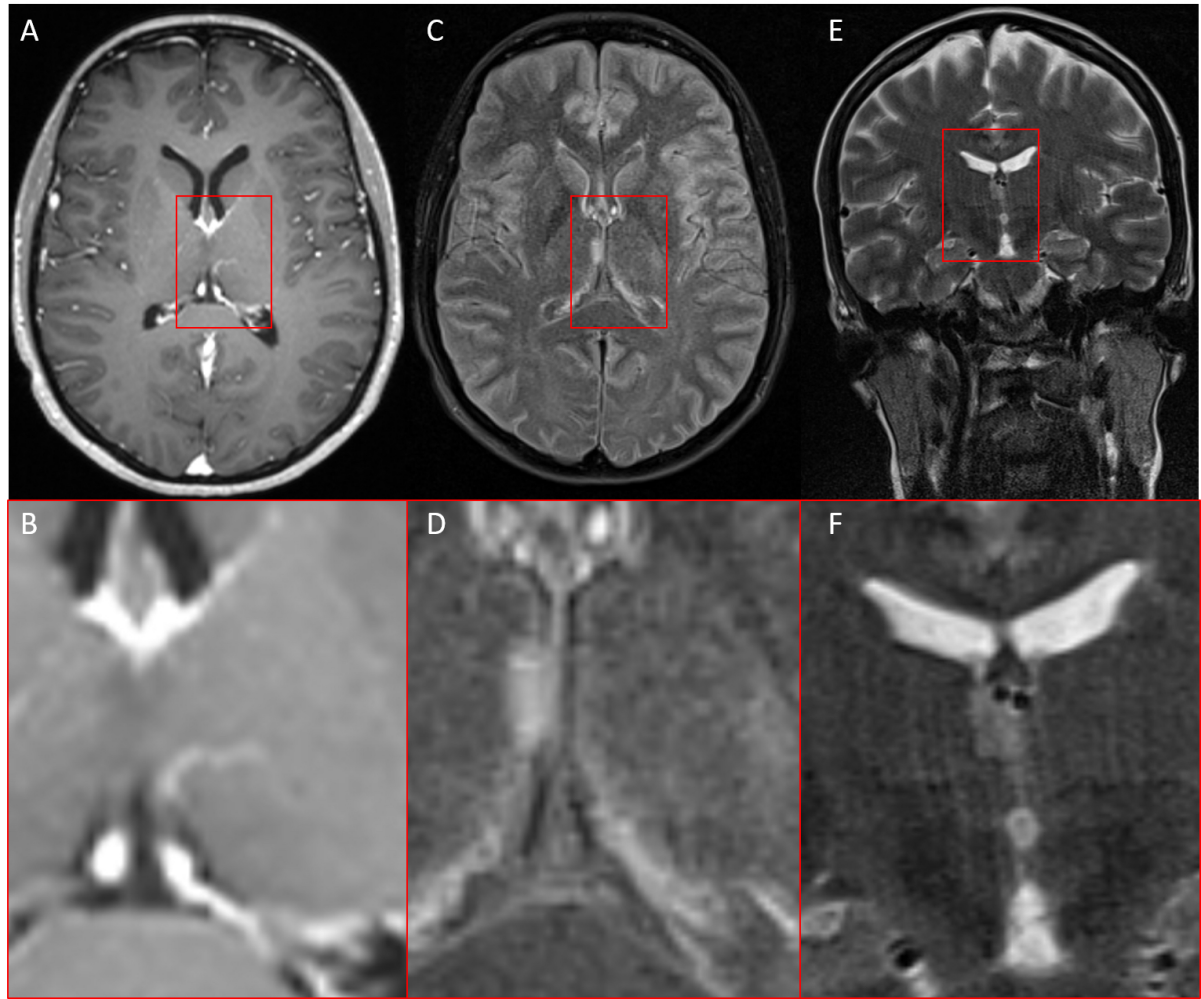
MRI of the same patient six years earlier. **(A, B)** T1w with contrast agent, **(C, D)** FLAIR (fluid-attenuated inversion recovery), **(E, F)** T2w coronar, In retrospect, a small FLAIR-/T2w-hyperintense lesion without contrast enhancement in the right thalamus adjacent to the third ventricle.

Elevated FLAIR Signal indicates high fluid component or high tissue density. Contrast enhancement was not visible, which suggests that there was no significant or detectable disruption of the blood-brain barrier at the time of imaging (**Figure 4**).

The differential diagnoses for the observed lesion presented a broad spectrum of possibilities. One potential explanation could be simple gliosis, possibly resulting from a prior ischemic event. An inflammatory origin should also be considered. The periventricular location of the lesion was particularly suggestive of this possibility. A vascular etiology could also account for the observed lesion. While dedicated vascular sequences were not available, the post-contrast T1 images showed no evidence of vascular malformation or pathological vessels.

### Postoperative course

Postoperatively, the patient developed meningitis and ventriculitis caused by Staphylococcus epidermidis 7 days after surgery. An endoscopic abscess cleaning was conducted 29 days after surgery, and a tunneled external ventricular drain (EVD) was placed. The patient underwent combined radiochemotherapy from day 65 to day 85, with a total dose of 40.05 Gy at 2.67 Gy per fraction, along with concurrent daily Temozolomide at 75 mg/m². From approximately 115 days to 300 days post-surgery, the patient received adjuvant chemotherapy with Temozolomide, starting with 150 mg/m² for the first cycle and 200 mg/m² for the second cycle. Due to thrombocytopenia and leukopenia, doses were reduced in subsequent cycles: 150 mg/m² for the third cycle, 135 mg/m² for the fourth and fifth cycles, and 145 mg/m² for the sixth cycle. Currently the patient presents in follow up MRI (441 days after resection) with stable disease RANO 2.0.

## Discussion and conclusion

With the introduction of the 5th edition of the WHO classification for CNS tumors 2021, it is now possible to distinguish between adult-type and pediatric-type gliomas by defined molecular markers (2). The pediatric high-grade glioma family can in turn be subdivided into four subtypes. One of them is the diffuse midline glioma H3K27 altered, which is mainly characterized by its location in midline structures (thalamus, mesencephalon, pons, cerebellum, and spinal cord). The prognosis is very poor, with often limited resectability given the tumor’s typical location and insufficient therapy options (10), which leads to a long-term survival of only a few months (15, 16). Although it is known that somatic point mutations in histone variants H3.3 and H3.1, leading to the exchange of lysine to methionine at amino acid position 27 (5, 6), are a primary driver event which leads to epigenetic dysregulation of oncogenes and tumor suppressor genes, only little is known about the further genetic evolution of these tumors. Although it is classified as a pediatric-type glioma, the age distribution ranges from the first years of life to old age (17, 18). In this case report we present a 38-year-old female patient with a molecularly confirmed diagnosis of a diffuse midline glioma H3K27 altered in the right thalamus. A retrospective review of an MRI from six years ago revealed a small, non-enhancing lesion adjacent to the third ventricle, presenting a broad differential diagnosis. Initially, the lesion was not described, and advanced MRI techniques such as perfusion and diffusion imaging were not performed. However, given the lesion’s small size and benign appearance, even these sophisticated methods might not have definitively established the diagnosis. Another diagnostic option could have been a biopsy of the lesion, which might have detected early histomolecular pathology. However, the lesion’s location in a highly vulnerable area of the brain presented significant risks. The potential for an unsuccessful biopsy or surgical complications would have been considerable, potentially outweighing the diagnostic benefits. In retrospect, the most prudent approach would have been to conduct follow-up examinations at regular intervals. This strategy would have allowed for the detection of any dynamic changes in the lesion over time. Such sequential imaging could have revealed subtle alterations in size, appearance, or surrounding tissue, potentially indicating the lesion’s true nature earlier in its development. The challenges in diagnosing this case are not unique, as H3K27-altered gliomas are extremely rare, with even large medical centers encountering only a few cases each year. Moreover, the official recognition of this diagnosis was only established in 2021 with the updated WHO Classification of Tumors of the Central Nervous System. This recent classification means that many earlier cases may not have been identified as H3K27-altered gliomas due to lack of specific testing or awareness. Given these diagnostic challenges and the rarity of reported cases, it is plausible to assume that the lesion observed six years prior was indeed a very early, potentially precursor lesion of the tumor. This observation challenges the assumption of a rapid *de novo* genetic evolution and growth typically associated with this tumor entity, which carries a median survival rate of only a few months, despite differences in individual age groups.

Interestingly, the patient remains in stable disease according to RANO 2.0 criteria approximately 14.5 months after resection, following standard treatment based on the Stupp protocol.

Both the tumor location and extended survival observed in this case align with recent literature findings (19). Schulte et al. demonstrated that adult H3K27M-mutant gliomas predominantly occur in midline structures, particularly the thalamus, and exhibit significantly better overall survival, averaging 27.6 months. This represents a marked improvement compared to historical data for both pediatric H3K27M-mutant diffuse midline gliomas and adult IDH-wildtype glioblastomas. Molecular analyses by Schulte et al. revealed that adult gliomas exclusively harbored mutations in the H3F3A gene, lacking mutations in HIST1H3B or HIST1H3C that are present in approximately one-third of pediatric diffuse midline gliomas (DMGs). In pediatric H3K27M gliomas, primarily located in the pons, tumor cells have been shown to exist in a self-renewing oligodendrocyte precursor cell (OPC) state, exhibiting oncogenic programs and stem cell-like profiles that contribute to their stable tumor-propagating potential (20). Liu et al. confirmed these findings but also demonstrated that this OPC state could be detected in thalamic gliomas. However, they observed cellular differences between pontine and thalamic tumors. Specifically, pontine H3-K27M DMGs were found to harbor more immature pre-OPC-like tumor cells compared to their thalamic counterparts as mesenchymal signature seems to increase with higher age, which was linked to age-related differences in tumor-associated macrophages (TAMs) (17).

However, a significant knowledge gap in molecular characteristics remains between adult and pediatric gliomas.

As these studies give insights to spatial tumor heterogeneity, the temporal evolution and which factors determine the malignant phenotype remains elusive.

Similar to glioblastomas, it is possible that these tumors develop years before they become symptomatic (21, 22). The mentioned study from Li et al. highlighted that these tumors, like glioblastoma, exhibit significant intratumoral genetic heterogeneity. Tumor progression is driven by clonal evolution, with subclones acquiring additional mutations over time, and is as such a stochastic process. This is evidenced by a highly subclonal architecture, where early driver mutations, including the hallmark H3K27M mutation, are present across all tumor cells, while later mutations (e.g., in TP53 or ACVR1) are subclonal and contribute to tumor adaptation. The molecular evolution of H3K27M tumors is therefore best characterized by a combination of early driver mutations (anchored by the H3K27M mutation) and subsequent adaptive changes driven by clonal selection and epigenetic reprogramming (17).

On the other hand it is also conceivable that the initial tumor was a low-grade tumor, such as a pilocytic astrocytoma with H3K27 mutation, which experienced a secondary malignant transformation, acquiring further mutations such as TP53 as described by Hochart et al. (12).

Especially in view of the fact that new therapeutic options have recently emerged like an effective long peptide vaccine targeting H3K27M (23), it would be of high interest to investigate the genetic pathway of these malignancies in order to potentially develop better individualized therapies to target tumorigenesis at an early stage.

## Limitation and perspective

This single case study challenges the current understanding of *de novo* evolution in H3K27-altered gliomas, suggesting a potentially longer evolutionary process. It highlights the need to reconsider the genetic and radiological evolution of high-grade gliomas, like evolving perspectives on classical glioblastomas. Understanding the factors determining tumor biology and malignant transformation could provide future targets for therapies. This case emphasizes the importance of longitudinal studies and comprehensive molecular profiling in glioma research. While this single case cannot serve as definitive proof, it acts as a catalyst for further research into the complex evolutionary pathways of H3K27-altered gliomas and other high-grade gliomas, potentially reshaping our approach to these challenging tumors.

## Data Availability

The data analyzed in this study is subject to the following licenses/restrictions: The genomic and imaging data is not available due to privacy restrictions. Requests to access these datasets should be directed to michael.griessmair@tum.de.
